# *Vochysia rufa* Stem Bark Extract Protects Endothelial Cells against High Glucose Damage

**DOI:** 10.3390/medicines4010009

**Published:** 2017-02-21

**Authors:** Neire Moura de Gouveia, Sonia Ramos, María Ángeles Martín, Foued Salmen Espindola, Luis Goya, Olga M. Palomino

**Affiliations:** 1 Department of Biofunctional Science, Faculty Morgana Potrich, Mineiros, Goias 75830-000, Brazil; neyrebio@yahoo.com.br; 2 Department of Metabolism and Nutrition, Institute of Food Science, Technology and Nutrition (ICTAN–CSIC), Madrid 28040, Spain; s.ramos@ictan.csic.es (S.R.); amartina@ictan.csic.es (M.Á.M.); luisgoya@ictan.csic.es (L.G.); 3 Institute of Genetics and Biochemistry, Federal University of Uberlandia, Uberlandia, Minas Gerais 38400-712, Brazil; fouedespindola@gmail.com; 4 Department of Pharmacology, Faculty of Pharmacy, Universidad Complutense de Madrid, Madrid 28040, Spain

**Keywords:** *Vochysia rufa*, Sapotaceae, endothelial cells, oxidative damage, plant antioxidants

## Abstract

**Background:** Increased oxidative stress by persistent hyperglycemia is a widely accepted factor in vascular damage responsible for type 2 diabetes complications. The plant *Vochysia rufa* (Vr) has been used in folk medicine in Brazil for the treatment of diabetes. Thus; the protective effect of a Vr stem bark extract against a challenge by a high glucose concentration on EA.hy926 (EA) endothelial cells is evaluated. **Methods:** Vegetal material is extracted with distilled water by maceration and evaporated until dryness under vacuum. Then; it is isolated by capillary electrophoresis–tandem mass spectrometry. Cell viability is evaluated on EA cells treated with 0.5–100 µg/mL of the Vr extract for 24 h. The extract is diluted at concentrations of 5, 10 and 25 µg/mL and maintained for 24 h along with 30 mM of glucose to evaluate its protective effect on reduced glutathione (GSH); glutathione peroxidase (GPx) and reductase (GR) and protein carbonyl groups. **Results:** *V. rufa* stem bark is composed mainly of sugars; such as inositol; galactose; glucose; mannose; sacarose; arabinose and ribose. Treatment with Vr up to 100 µg/mL for 24 h did not affect cell viability. Treatment of EA cells with 30 mM of glucose for 24 h significantly increased the cell damage. EA cells treated with 30 mM of glucose showed a decrease of GSH concentration and increased Radical Oxygen Species (ROS) and activity of antioxidant enzymes and protein carbonyl levels; compared to control. Co-treatment of EA with 30 mM glucose plus 1–10 μg/mL Vr significantly reduced cell damage while 5–25 μg/mL Vr evoked a significant protection against the glucose insult; recovering ROS; GSH; antioxidant enzymes and carbonyls to baseline levels. **Conclusion:** *V. rufa* extract protects endothelial cells against oxidative damage by modulating ROS; GSH concentration; antioxidant enzyme activity and protein carbonyl levels.

## 1. Introduction

The oxidation-reduction reactions are physiological processes aimed to release different substances which are needed in cellular metabolism. These reactions involve the transfer of electrons and may generate compounds known as reactive oxygen species (ROS) such as superoxide anion (O_2_^−^), hydroxyl (·OH) and peroxyl (ROO·) which are oxygen derived molecules with high reactivity. Oxidative stress is defined as the imbalance between the production of free radicals and the ability of a biological system to quickly detoxify the reactive intermediates and repair the damage caused at protein, lipid and DNA levels which induce different diseases [1]. 

One of the first affected tissues is the vascular endothelia exposed itself to blood, this being the first step towards the development of vascular diseases, such as hypertension and atherosclerosis [2]. Oxidative stress may cause functional disorders of vascular endothelia and thus alter the function and structure of the vascular tissues [3]. In fact, damage to vascular endothelium is the primary complication of type 2 diabetes mellitus leading to endothelial dysfunction and further complications such as diabetic nephropathy [4]. Therefore, prevention of oxidative stress is one of the main objectives of nowadays cardiovascular research, natural products being a source of active substances with promising applications [5]. Several studies demonstrate that plant antioxidants protect the endothelium against oxidative stress and then become an effective option to treat vascular diseases [6,7].

*Vochysia rufa* Mart belongs to the Vochysiaceae family and grows as a neotropical tree in Brazil [8]. The seed of *V. rufa* is recognized for its nutritional value, due to its content in proteins, sugars, oil and fatty acids, mainly stearic and oleic acids [9]. Into the seed cell walls, arabinose, galactose and glucose have been shown as the most abundant constituents, followed by mannose and rhamnose. The polyssacharide composition of seeds from this family contains a mixture of arabinogalactan and mannan which is similar to soluble coffee carbohydrate composition [10]. The bark of the plant is known as sweet bark and has been used in folk medicine for the treatment of diabetes mellitus in the area of Uberlandia (Brazil). Few studies also referred to the presence of polyphenolic compounds such as coumarins or saponins, together with terpenoids, within this genus [11,12] with no reference to the carbohydrates content of the bark.

Since hyperglycemia-induced oxidative damage to endothelial cells is a main cause of micro- and macro-vascular complications of type 2 diabetes and the hypoglycemic effect and the attenuation of oxidative stress in hepatic tissue of streptozotocin (STZ)-induced diabetic rats treated with Vr has been documented [13], the objective of this study was to test the chemo-protective effect of a Vr extract on endothelial cells submitted to high glucose concentrations. Thus, in this study a human endothelial cell line, EA.hy926, was used as a cell culture model of endothelium and in vitro treatment with high glucose was used to reproduce an in vivo condition of hyperglycemia-induced oxidative stress in order to study the possible protective mechanisms exerted by a *Vochysia rufa* on endothelial function. 

## 2. Materials and Methods 

### 2.1. Reagents and Materials

*Tert*-butylhydroperoxide (*t*-BOOH), (−)-epicatechin (EC, >95% of purity), Glutathione reductase (GR), reduced glutathione (GSH) and oxidized glutathione, dichlorofluorescin (DCFH), *o*-phthaldialdehyde (OPT), nicotine adenine dinucleotide phosphate reduced salt (NADPH), gentamicin, penicillin G and streptomycin were purchased from Sigma-Aldrich Chemical (Madrid, Spain). Bradford reagent was from BioRad Laboratories S.A (Madrid, Spain). Methanol of HPLC grade, dimethyl sulfoxide (DMSO), light petroleum and phosphoric acid of analytical grade were purchased by Panreac (Barcelona, Spain). DMEM culture media and foetal bovine serum (FBS) were from Cultek (Madrid, Spain). All other reagents were of analytical quality.

### 2.2. Plant Material

Samples of *V. rufa* stem bark collected from the Cerrado biome (Brazil) were identified and a voucher specimen was deposited at the Herbarium Uberlandensis of the Universidade Federal de Uberlandia with voucher number 58,888.

The bark was dried at 40 °C and ground to a powder. Then it was extracted by maceration for 24 h at room temperature, filtered and centrifuged at 2000× *g* at 4 °C for 15 min. Finally, the supernatant was collected, frozen and lyophilized.

### 2.3. Quantification of Reducing Sugars

The presence of reducing sugars was determined according to the Lane-Eynon method which is based in the reduction of cupric salts in alkaline tartrate solution by heating aldoses and ketoses which turn into red cuprous salts [14]. Briefly, solutions A and B of Fehling were led to boiling point, together with distilled water. Then, the test sample was transferred to a burette and added dropwise over Fehling’s solution, boiling, with continuous stirring until the solution changed from blue to colorless. A reddish residue was formed in the flask bottom; methylene blue was added and the titration was completed according to the change of color. A solution of the aqueous extract of 0.5% was prepared and carried out the same procedures to glucose. Sugars were isolated by capillary electrophoresis–tandem mass spectrometry [15].

### 2.4. Cell Culture

The human hybrid cell line EA.hy926 was supplied by Profs. Patricio Aller and Carmelo Bernabeu, from the Biological Research Centre (CIB, CSIC, Madrid, Spain). The cell line was cultured and passaged in Biowhittaker DMEM media supplemented with 10% fetal bovine serum. Cells were maintained in a humidified incubator containing 5% CO_2_ and 95% air at 37 °C and grown in DMEM medium supplemented with 10% FBS and 50 mg/L of each of the following antibiotics: gentamicin, penicillin and streptomycin. The culture medium was changed every other day in order to remove the not adherent and dead cells and the plates were usually split 1:3 when they reached confluence. No human subjects, material or data used. 

### 2.5. Cell Treatment with EC or Vr 

EC, used as a positive control for the experiments, was dissolved in 50% methanol and diluted in DMEM media. A Vr extract stock solution of 10 mg/mL in distilled water was prepared and stored at −20 °C. Stock solution was diluted the day of the experiment with FBS-free DMEM medium to prepare the concentrations to test. To assay the direct effect of the extract cells were incubated with the noted concentrations for 24 h. As previously reported [16], to induce a condition of damage by oxidative stress, EA.hy926 cells were treated with 30 mM glucose for 24 h and then tested for antioxidant defenses and carbonyl groups. To evaluate the protective effect of Vr against high glucose-induced toxicity, concentrations of the extract (1, 5, 10 or 25 µg/mL) were added together with 30 mM glucose to the cell plates for 24 h.

### 2.6. Evaluation of Cell Viability and ROS Production

Cell viability was determined by using the crystal violet assay [17]. Concentrations of Vr ranging from 0.5 to 100 µg/mL were diluted in DMEM culture medium and added to the cell plates for 24 h to test the direct effect of the extract. The same doses of the Vr extract were used as co-treatment with glucose to assay the protective effect. Cells were seeded at low density (10,000 cells per well) in 96-well plates, grown for 24 h and incubated with crystal violet (0.2% in ethanol) for 20 min. Plates were rinsed with water and 1% sodium dodecylsulphate added. The absorbance of each well was measured using a microplate reader at 570 nm. Intracellular ROS were quantified by the DCFH fluorometric assay using a micro plate reader [18]. After being oxidized by intracellular oxidants, DCFH becomes dichorofluorescein and emits fluorescence. Cells were cultured in 24-wells multiwall, treated for 2 h with 5 µM EC or different concentrations of Vr (5, 10, 25, 50 µg/mL) and then the DCFH probe was added for 30 min, the unabsorbed probe was removed and fluorescence at 485 nm/530 nm determined to evaluate the direct effect. To assay the protective effect cells were treated with noted concentrations of EC or Vr for 24 h, then submitted (except controls) to 30 mM glucose for 2 h and ROS were quantified as above.

### 2.7. Determination of GSH Concentration and GPx and GR Activity

The protective effect of 5–25 µg/mL Vr extract on antioxidant defenses was evaluated in EA.hy926 cells submitted to 30 mM glucose. The concentration of GSH was evaluated by a fluorometric assay previously described [18]. The method takes advantage of the reaction of GSH with OPT at pH 8.0 and fluorescence was measured at an emission wavelength of 460 nm and an excitation wavelength of 340 nm. The determination of GPx activity is based on the oxidation of reduced glutathione by GPx, using *t*-BOOH as a substrate, coupled to the disappearance of NADPH by GR. GR activity was determined by following the decrease in absorbance due to the oxidation of NADPH utilized in the reduction of oxidized glutathione [18]. Protein was measured by the Bradford reagent.

### 2.8. Determination of Carbonyl Groups

The protective effect of 5–25 µg/mL Vr extract on a specific biomarker of protein damage was evaluated in EA.hy926 cells submitted to 30 mM glucose Protein oxidation of cells was measured as carbonyl groups content in supernatants according to a published method [19]. Absorbance was measured at 360 nm and carbonyl content was expressed as nmol/mg protein using an extinction coefficient of 22,000 nmol/L/cm. Protein was measured by the Bradford reagent.

### 2.9. Statistics

Statistical analysis of data was as follows: prior to analysis the data were tested for homogeneity of variances by the test of Levene; for multiple comparisons, one-way ANOVA was followed by a Bonferroni test when variances were homogeneous or by Tamhane test when variances were not homogeneous. The level of significance was *p* < 0.05. A SPSS version 22.0 program has been used.

## 3. Results

### 3.1. Quantification of Reducing Sugars

*V. rufa* stem bark is rich in reducing sugars as the extract contained 70% reducing carbohydrates. Inositol, galactose, glucose and mannose with *m*/*z* of 179; sacarose with *m*/*z* of 341; arabinose and ribose with *m*/*z* of 149 have been previously identified in the extract. LC-MS analyses also suggested the presence of phenolic compounds of flavonoid type [15,20].

### 3.2. Cell Viability and ROS Production

Treatment with concentrations ranging from 0.5 to 100 µg/mL of Vr extract during 24 h induced no cell damage on EA.hy926 cells, as no variation in crystal violet staining was observed (Table 1). Treatment of cells with 30 mM glucose evoked a massive decrease in cell viability (around 50%) that was partially (1 µg/mL) or completely (5–100 µg/mL) abolished by a co-treatment with Vr extract or EC (Table 1). As with cell viability, treatment with Vr did not affect ROS concentration indicating no cellular stress or oxidative damage which could influence the functional conditions of cells to face stressful injury (Table 2). When EA.hy926 cells were submitted to 30 mM glucose for 2 h a dramatic increase in ROS production was observed, but this increase was partially avoided by a co-treatment with 5–10 µg/mL Vr extract, whereas a complete recovery of ROS was observed with 25–50 µg/mL Vr and EC (Table 2).

### 3.3. GSH Concentration and GPx and GR Activity

**Figure 1:**
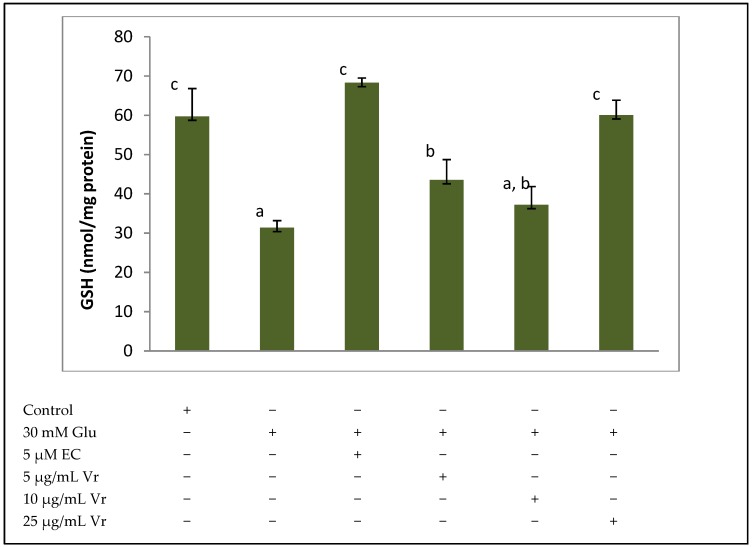
Protective effect of *Vochysia rufa* bark extract on GSH levels of EA.hy926 cells treated with 30 mM glucose (Glu 30 mM) or 30 mM glucose plus noted concentrations of *Vochysia rufa* (Vr) extract or the positive control Epicatechin (EC). Values are means ± SD, *n* = 4. Values are expressed as nmol GSH/mg protein. Different letters indicate statistically significant differences (*p* < 0.05) among groups.

The protective effect of 5–25 µg/mL Vr extract on GSH concentration and GPx and GR activities was evaluated in EA.hy926 cells submitted to 30 mM glucose. A dramatic depletion (about 50%) of intracellular GSH levels was observed when 30 mM glucose was added for 24 h to EA.hy926 cells; however, co-treatment with 5 μM EC or 25 μg/mL of extract completely prevented the depletion of GSH induced by high glucose, whereas a partial recovery of GSH was observed with 5 μg/mL (Figure 1).

**Figure 2:**
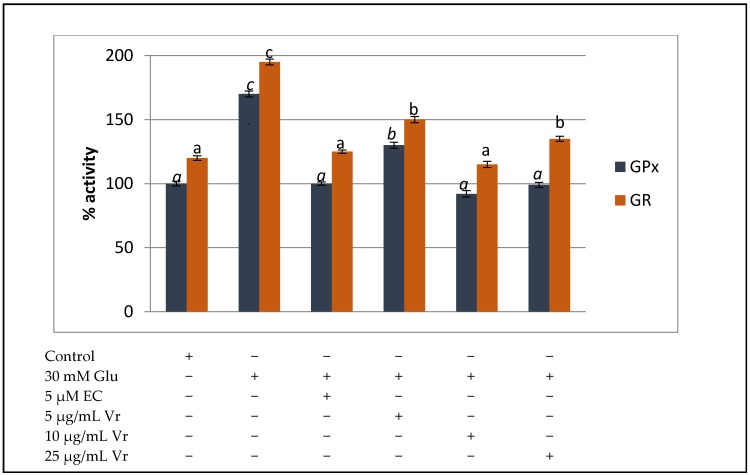
Protective effect of *Vochysia rufa* bark extract on GPx and GR activity of EA.hy926 cells treated with 30 mM glucose (Glu 30 mM) or 30 mM glucose plus noted concentrations of *Vochysia rufa* (Vr) extract or the positive control Epicatechin (EC). Values are means ± SD, *n* = 4. Values are expressed as a percent relative to activity of control condition. Different letters indicate statistically significant differences (*p* < 0.05) among groups. Letters in italics correspond to statistical significance in GPx.

Treatment of EA.hy926 cells with 30 mM glucose evoked a significant increase of GPx and GR activities in order to face the glucose challenge. Co-treatment of endothelial cells submitted to high glucose with 5–25 µg/mL of Vr extract efficiently returned GPx and GR activities to basal values preparing cells to further oxidative insults, in a comparable way than the positive control, EC (Figure 2).

### 3.4. Carbonyl Groups

**Figure 3:**
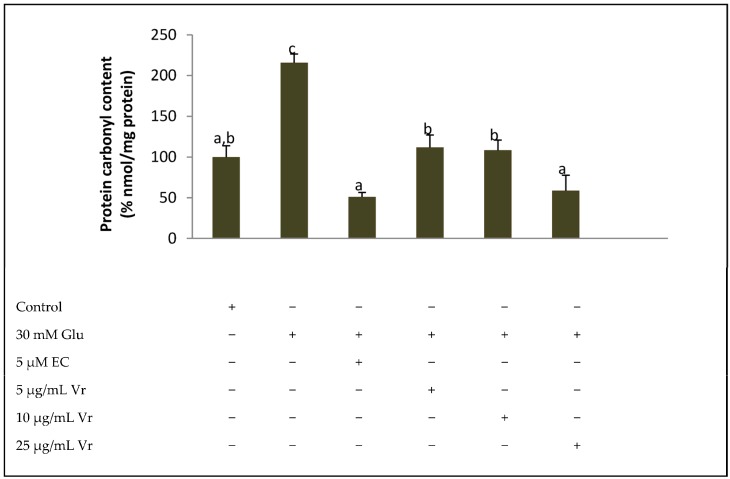
Protective effect of *Vochysia rufa* bark treatment on carbonyl group production by EA.hy926 cells. Content of carbonyl groups was evaluated in EA.hy926 cells treated with 30 mM glucose (Glu 30 mM) or 30 mM glucose plus noted concentrations of *Vochysia rufa* (Vr) extract or the positive control epicatechin (EC). Values are means ± SD, *n* = 3. Values are expressed as a percent relative to control condition. Different letters indicate statistically significant differences (*p* < 0.05) among groups.

One of the most consistent biomarkers of oxidative damage to proteins is carbonyl groups [21]. The significant increase in carbonyl groups in EA.hy926 cells treated with high glucose concentrations (30 mM) indicates an extensive damage to cellular proteins at cellular (Figure 3). Co-treatment of cells with 5 µM EC or 5, 10 and 25 µg/mL Vr extract significantly reduced the percentage of carbonyl groups in response to high glucose, demonstrating a decreased protein oxidation under a stressful situation (Figure 3).

## 4. Discussion

*V. rufa* stem bark is rich in carbohydrates, mainly reducing sugars such as inositol, galactose, glucose, mannose, sacarose, arabinose and ribose. Additionally, the presence of polyphenols and triterpenes has been reported in other *Vochysia* species [9,11]. The in vivo hypoglycemic effect of polysaccharide has been reported for other species such as *Coptis chinensis* or *Taxus cuspidata*, where an efficient antidiabetic effect and antioxidant activity on diabetic mice was observed [22,23]. Thus, the protective antioxidant activity obtained on endothelial cells may be due not only to the presence of phenolics but also to the carbohydrates previously identified which improved the antioxidant enzymes activity and the elevated lipid peroxidation in diabetic rats [13]. 

The flavan-3-ol EC, previously identified in cocoa and tea, among other edible herbs, has proved a powerful protective compound against a chemically-induced oxidative stress in hepatic [21], colonic [24] and pancreatic beta [18] cells. Thus, EC was used as a positive control in assays of protective effect to evaluate and compare the chemo-protective effect of Vr. The concentration range used to study the effect of Vr on viability and redox status of EA.hy926 cells is not far from realistic in order to evaluate the effect at the biological level. Thus, concentrations within the μM range of some pure compounds have been reported in rat plasma after the consumption of equivalent doses of different plant extracts [16,21]. The carbohydrate-rich Vr extract did not affect cell viability or ROS production in endothelial whereas the treatment of the cells with 30 mM of glucose for 24 h significantly increased generation of ROS and, consequently, the cell death. Vr concentrations from 1 to 100 µg/mL significantly reduced cell damage evoked by glucose, and the effect of 5–100 µg/mL Vr was comparable to that of EC. In line with this viability protection, doses of 10–50 µg/mL Vr extract considerably reduced ROS levels in an equivalent capacity than EC, indicating that the presence of the Vr extract in the culture medium prevents the harmful increase of ROS and the imbalance of cellular redox status and, consequently, precludes cell death. A similar chemo-protective effect has been previously reported for other plant extracts in different cultured cells [16,21,24].

Reduced Glutathione (GSH) is the main non-enzymatic antioxidant defence as a substrate in glutathione peroxidase-catalysed detoxification of organic peroxides, by reacting with free radicals and by repairing free radical induced damage through electron-transfer reactions. It should be emphasized that the loss of cellular GSH seems to have an important role in apoptotic signalling [25]. Therefore, maintaining GSH concentration above a critical threshold while facing a stressful situation represents a crucial advantage for cell survival. While 5 and 10 µg/mL Vr evoked a partial protection against the glucose insult, 25 µg/mL Vr and 5 μM EC fully recovered GSH to baseline levels. We have recently reported a similar GSH recuperation with equivalent concentrations of an aqueous extract of *Silybum marianum* [16]. Additionally, other studies have reported comparable recoveries of GSH with antioxidant extracts from cocoa [24,26], and cranberry [21] in different cell types submitted to oxidative stress. Vr stem bark contains a relevant amount of reducing sugars such as arabinose, galactose, glucose as well as mannose and rhamnose, which are capable of acting as reducing agents, because of the free aldehyde or ketone groups that may provide H+ to regenerate GSH. Although the antioxidant effect of a reducing sugar will not reach the potency of the powerful flavonoid EC, the synergic effect of different reducing sugars and other components of the extract may surely be capable of help the endothelial cells to recover GSH.

The enzymatic constituents of antioxidant defence system play a crucial role against oxidative stress; thus, the significant increase in the activity of GPx and GR observed after 24 h treatment with 30 mM glucose clearly indicates a positive response of the cell defence system to face the exposure to high glucose and overcome the oxidative insult. However, a rapid return of the antioxidant enzyme activities to basal values once the challenge has been surmounted will position the cell in a favorable condition to deal with a new glucose challenge [16,26]. The present study has shown that, similar to what observed in cells treated with the positive control EC, co-treatment of high glucose-submitted endothelial cells with 10–25 µg/mL of Vr extract can efficiently return GPx and GR activities to basal values, enabling cells in to face further oxidative insults. Accordingly, we have previously reported that a realistic treatment with an antioxidant-rich cocoa extract averted cell damage by preventing the permanently increased activity of GPx and GR induced by high glucose in liver cells [26] and diabetic fatty rats [27]. More recently, we have also shown a similar recovery of antioxidant defences in EA.hy926 cells submitted to the same high glucose challenge with an extract from *Silybum marianum* [16]. These results, together with those of GSH, indicated that the prevention or delay of appearance of conditions causing oxidative stress in the cell may also reflect the ability of a compound to modulate the cellular antioxidant defences.

One of the most consistent biomarkers of oxidative damage to proteins is carbonyl groups [26,28]. The significant increase in carbonyl groups in EA.hy926 cells treated with high glucose concentrations (30 mM) indicating an extensive damage to cellular proteins has been recently established. Co-treatment of cells with 5 μM EC or 5, 10 and 25 μg/mL Vr extract significantly reduced the percentage of carbonyl groups in response to high glucose, demonstrating a decreased protein oxidation under a stressful situation. Similarly, administration of comparable concentrations of *S. marianum* extract to EA.hy926 cells submitted to the same high glucose challenge significantly reduced levels of carbonyl groups [16]. In the same line, an equivalent chemo-protective effect on carbonyl groups has also been reported in high-glucose challenged hepatic cells treated with cocoa flavonoids [26], and in a condition of hyperglycaemia in livers from diabetic rats fed a diet rich in cocoa [27].

Thus, the protective mechanism of Vr extract on high glucose-damaged endothelial cells can be illustrated in terms of regulation of the cellular redox status. The Vr extract is rich in reducing sugars and phenolic antioxidants which moderate the production of oxygen radicals and regenerate GSH; these two effects together help to reduce the expense of GSH and prevent a dangerous decrease of its cellular concentration. Once GSH concentration is maintained at safe values, there is no need for GR to be activated for long time and can return to a latent activity; besides, the diminished generation of ROS by the reducing agents of Vr evokes a smaller oxidative damage to proteins (carbonyl groups) and prevents cell death. This group of results indicates that integrity of EA.hy926 cells treated with Vr extract was protected against the oxidative insult induced by high glucose.

It is worth mentioning that since the Vr extract is a mixture of many different components, many of them phytochemicals with reducing potential, a synergistic effect of all of them should not only be discarded but expected. Along with a fair amount of reducing sugars, other studies have reported the presence of polyphenolic compounds such as coumarins or saponins, together with terpenoids, all phytochemical compounds with antioxidant capacity and available for a synergistic effect to protect the cell against a stressful situation.

## 5. Conclusions

*Vochysia rufa* stem bark is rich in carbohydrates, mainly reducing sugars and some antioxidant phytochemicals. This work shows that realistic doses of *V. rufa* contribute to the protection of endothelial cells submitted to oxidative damage by high glucose via modulating GSH concentrations and antioxidant enzyme activity and maintaining ROS and protein carbonyl levels at safety levels. Considering all data, it can be concluded that treatment of EA.hy926 with *V. rufa* practically normalizes antioxidant defense system of endothelial cells in spite of the oxidative challenge. Further experiments are needed to assess and define the mechanism of action of this biological activity in experimental animals previous to the potential test in humans in order to confirm *Vochysia rufa* extract as a potential therapeutics in this field.

## Figures and Tables

**Table 1:** Direct and protective effect of *Vochysia rufa* treatment on EA.hy926 cell viability. Cell viability was evaluated in EA.hy926 cells treated for 24 h with noted concentrations of *Vochysia rufa* (Vr) extract (direct effect). To assay the protective effect cells were treated for 24 h with 30 mM glucose (30 mM Glu) or 30 mM glucose plus noted concentrations *Vochysia rufa* (Vr) extract or the positive control epicatechin (5 µM EC). Values are means ± SD. *n* = 8 (cell viability). Values are expressed as a percent relative to control condition. Different letters in each column indicate statistically significant differences (*p* < 0.05) among groups.

Condition	Cell Viability ± SD	
	Direct Effect	Protective Effect (+30 mM Glu)
Control	100.37 ^a^ ± 1.34	100 ^c^ ± 5.17
30 mM Glu	-	54.80 ^a^ ± 1.29
5 µM EC	-	115.1 ^c^ ± 11.0
0.5 µg/mL *V. rufa*	99.06 ^a^ ± 1.83	57.85 ^a^ ± 4.39
1 µg/mL *V. rufa*	99.59 ^a^ ± 1.12	66.93 ^b^ ± 3.53
5 µg/mL *V. rufa*	100.08 ^a^ ± 2.22	123.64 ^d^ ± 3.48
10 µg/mL *V. rufa*	99.15 ^a^ ± 1.35	123.31 ^d^ ± 3.94
25 µg/mL *V. rufa*	99.96 ^a^ ± 1.52	126.50 ^d^ ± 2.09
50 µg/mL *V. rufa*	99.05 ^a^ ± 1.24	122.16 ^d^ ± 3.61
100 µg/mL *V. rufa*	98.23 ^a^ ± 1.03	108.48 ^c^ ±.6.10

**Table 2:** Direct and protective effect of *Vochysia rufa* treatment on ROS generation in EA.hy926. ROS generation was evaluated in EA.hy926 cells treated for 2 h with noted concentrations of EC or the *Vochysia rufa* (Vr) extract (direct effect). To test the protective effect cells were treated for 24 h with noted concentrations of EC or *Vochysia rufa* (Vr) extract and then submitted to 30 mM glucose (30 mM Glu) for 2 h. Values are means ± SD. *n* = 4 (ROS). Values are expressed as a percent relative to control condition. Different letters in each column indicate statistically significant differences (*p* < 0.05) among groups.

Condition	ROS ± SD	
	Direct Effect	Protective Effect (+30 mM Glu)
Control	100 ^a^ ± 8.64	100 ^a^ ± 3.48
5 µM EC	86.02 ^b^ ± 2.25	113.27 ^b^ ± 3.80
30 mM Glu	-	298.07 ^d^ ± 6.30
5 µg/mL *V. rufa*	98.00 ^a^ ± 9.17	214.91 ^c^ ± 4.97
10 µg/mL *V. rufa*	103.63 ^a^ ± 6.02	122.74 ^b^ ± 4.74
25 µg/mL *V. rufa*	100.68 ^a^ ± 8.21	94.11 ^a^ ± 0.74
50 µg/mL *V. rufa*	95.96 ^a^ ± 9.20	91.63 ^a^ ± 1.55
